# On the Throat as the Source of Systemic Infection in Acute Rheumatism

**Published:** 1904-09

**Authors:** P. Watson Williams

**Affiliations:** Physician in charge of the Throat Department, Bristol Royal Infirmary; Lecturer on Practical Medicine, University College, Bristol


### ON THE THROAT AS THE SOURCE OF SYSTEMIC INFECTION IN ACUTE RHEUMATISM.

BY

P. Watson Williams, M.D. Lond.,

*Physician in charge of the Throat Department, Bristol Royal Infirmary; Lecturer on Practical Medicine, University College, Bristol.*

If acute rheumatism (by which term I refer only to that affection that is now very generally regarded as a disease *sui generis*, variously designated as "true rheumatism," "rheumatic fever," "acute rheumatism," &c.) is an infective disease due to a specific micro-organism, it follows that there are portals by which the infecting agent gains entrance to the tissues, and it seems probable that the throat furnishes that point of entry in a considerable, if not the most considerable, percentage of patients.

I am assuming that acute rheumatism depends upon an infective agent, a view which is supported by evidence cited[1] by Cheadle and Church, Hirsch, Newsholme (*Milroy Lectures*,

[1] Allbutt's *System of Medicine*, vol. iii., 1897, p. 4.

1895), Friedländer (cited by Newsholme[1]), &c., &c.; while the clinical and experimental investigations of Triboulet, Wassermann, and Coyon, corroborated and extended by Poynton and Paine[2] and by Beaton and Walker,[3] render it exceedingly probable that the non-capsulated micrococcus which has been isolated and grown from the tonsil, spleen, kidney, lung, and cardiac valvular vegetations is the specific infective agent so long sought.

I may refer to Poynton's last communication, which was published[4] on the 14th of May, describing the results of a bacteriological investigation of a fatal case of chorea and rheumatism. Cultures inoculated in rabbits and monkeys produced the lesions of rheumatic fever, *e.g.* arthritis, pericarditis, endocarditis, and from the tissues of the animals the diplococcus was again isolated in pure culture. Referring to the relation of tonsillitis to rheumatic fever, Poynton states that "it is certain that one channel of infection is through the damaged tonsils, in which the micro-organism is deposited." (P. 1118.)

The relation of acute tonsillitis to acute rheumatism has been recognised for many years. Trousseau,[5] for instance, in 1865 describes the rheumatic sore throat, and goes on to say that "all these phenomena are going to disappear with great rapidity, because they are fugacious, like most affections of a rheumatic nature. The next day . . . another pain will occupy the neck . . . ; whilst the day after, one of the shoulders may be the part attacked. . . . Patients who have had several attacks of this kind of sore throat are able at the outset to distinguish the rheumatic affection [of the throat] from a veritable phlegmonous affection." Mackenzie states that "in many rheumatic patients the throat affection is an invariable precursor of a general attack of sub-acute rheumatism."

It is not enough to show that tonsillitis and rheumatic fever are associated. My contention is that they are frequently interdependent. One of the many examples of the infectious nature

1 *Loc. cit.* 2 *Lancet*, 1900, ii. 861; *Brit. M. J.*, 1904, i. 1117 *et seq.*
3 *Brit. M. J.*, 1903, i. 237. 4 *Brit. M. J.*, 1904, i. 1117.
5 *Clin. Méd. de l'Hotel Dieu*, Paris, 1865, t. l., p. 332; cited by Mackenzie, *Dis. of Throat and Nose*, vol. i., 1880, p. 46

of true rheumatic tonsillitis, and therefore of acute rheumatism, is recorded by Lawson.[1] A healthy boy, after bathing in a river, had a moderate attack of tonsillitis, and a week later developed acute rheumatism from which he slowly recovered. His companion, a strong man, sleeping in an adjoining bed, developed tonsillitis ten days after the boy's illness began, and though he had no rheumatic family history, he too, ten days later, developed acute rheumatism and was ill for six weeks. Both subsequently developed serious cardiac symptoms.

Three cases of acute rheumatism in which the affection was spread from one to the other by tonsillitis are recorded by Allaria.[2] The cocci from the tonsils caused arthritis when injected into guineapigs.

That simple acute follicular tonsillitis is contagious can scarcely be questioned. Of this I have cited evidence in the last edition of my *Diseases of the Upper Respiratory Tract*, and it would be easy to multiply examples; but I will not "flog dead horses." Many instances of cardiac complications arising in the course of acute tonsillitis without even any arthritic symptoms of rheumatism have also been recorded.[3]

Some years ago I endeavoured to tabulate observations on the relation between enlarged tonsils and rheumatism, but I found that in a large number of cases of rheumatic fever, even when there was a history of preceding sore throat, there was not only no hypertrophy of the tonsils but often the tonsils were abnormally small or atrophied. I came to the conclusion that when tonsillitis is associated with marked hypertrophy of the tonsils, with or without enlargement of the cervical glands, there is less tendency to the development of systemic rheumatism than in patients without these hypertrophic changes; in other words, *that pronounced local inflammatory reaction is protective, while the slighter forms of tonsillitis without resulting lymphoid tissue hypertrophy are more prone to be the precursors of acute systemic infection*, and thus it seemed futile to hope for any reliable data on this particular point from statistics. This, after all, accords with

[1] *Brit. M. J.*, 1900, i. 1284.

[2] *Riv. crit. di clin. Med.*, November 23rd, 1901; cited in *Med. Ann.*, 1903, p. 581.

[3] F. A. Packard, *Am. J. M. Sc.*, 1900, cxix. 1.

the observations of Trousseau and Mackenzie on the fugacious tendency of what they regarded as true rheumatic sore throat.

Do we not observe a precisely similar relationship between tuberculous disease of the tonsils and cervical glands, for, though tuberculous adenitis with or without subsequent suppuration is a very common disease of childhood, it is comparatively distinctly rare to find evidence of former tuberculous adenitis in pulmonary tuberculosis?

I do not wish to suggest that all cases of acute tonsillitis are true local rheumatism, but that the majority are is rendered probable from the very frequent association of mild rheumatic symptoms and from the remarkable controlling effect of salicylates on the course of the tonsillitis, while in a considerable percentage the local tonsillitis is but the precursor of severe and typical rheumatic fever.

In introducing a discussion in the laryngological section of the British Medical Association meeting at Swansea last year de Havilland Hall said: "I think there is a great deal to be said in favour of the view that tonsillitis is a primary infective disease of the lacunæ, and that rheumatic fever is a secondary disease arising from the absorption of microbes or their products into the system . . . and that in a large number of cases the infection enters the system through Waldeyer's pharyngeal ring, especially the tonsils."

"We have no certain knowledge of the physiological functions of the faucial, lingual and pharyngeal tonsils; but Philip Stöhr has drawn attention to the fact that in their epithelial covering are gaps large enough to allow the passage of leucocytes. . . . Whilst the fissures and crypts of the tonsil form convenient resting-places or 'traps' for microbes, the peculiar anatomical arrangement of their epithelial covering opens the gates to their invasion."[1] Thus the upper respiratory tract is inevitably most prone to the chances of inoculation by any infective agent conveyed by air, and this we already recognise as affording the usual portal of infection by diphtheria largely because we observe the very plain indications of such

[1] Felix Semon and Watson Williams, Allbutt's *System of Medicine*, 1897, iv., 769, 770.

primary infection in this disease. The primary sore throat that is so characteristic of scarlatina is probably the initial lesion and site of invasion of the infective micro-organism, and whether that be the streptococcus of Klein and Gordon, the streptococcus conglomeratus of Kurth, or diplococcus of Class, the early appearance of the coccus in the fauces, tonsils or nasal mucosa, together with the clinical manifestations which mark the course of the disease, warrant our regarding the sore throat as the primary lesion of scarlatina just as a chancre on the penis is usually, though not invariably, the initial lesion and portal of entry of the virus in syphilis.

The readily-identified Klebs-Loeffler bacillus has enabled us to demonstrate that not only is the nose and throat the usual seat of invasion of diphtheria, but that these regions often harbour the bacillus after the clinical symptoms have subsided, and constitute a source of danger to the patient and to those who come in contact with him. It is becoming more generally recognised that the same holds good in scarlatina; and at the last annual meeting of the Incorporated Society of Medical Officers of Health, the medical officer for Swindon stated his opinion that return cases of scarlatina were due to the infectivity of mucous discharges from the nose and throat and ear, and not to desquamation, the infectivity of which was very doubtful. With these views I heartily concur, but I would suggest that in acute rheumatism also we must look to the fauces, tonsils, pharynx, larynx and nose as the common site of invasion, and that the infective agent generally persists in these regions long after the acute symptoms have disappeared. Park and others have shown that staphylococci and streptococci increase greatly in the mouth in virulence and number in damp weather and winter months. Hence we see how climatic conditions determine the virulence and pathogenic activity of these micro-organisms, probably always present in the fauces and tonsils of the recurrent rheumatic. Indeed, it is somewhat difficult to see why otherwise rheumatism should be so responsive to climatic changes.

I submit then that—

1. Acute rheumatism is an infective disease *sui generis*.

2. There is a true rheumatic pharyngitis and tonsillitis.

3. That rheumatic pharyngitis or tonsillitis is a primary infectious disease.

4. That rheumatic fever is a secondary infection, due either to the absorption of the products of the infective micro-organisms or to the growth of such micro-organisms in the tissues, and that the infection may manifest itself in arthritis, pericarditis, endocarditis, chorea, bronchitis, pleurisy, alone or in association.

5. That in a large percentage of cases the portal of infection is in the fauces or pharynx or other region of the upper respiratory tract, but most frequently the oro-pharyngeal lymphoid ring.

6. That there is no proportion between the intensity of the primary local lesion and the appearance or severity of the secondary systemic complications.

